# Pollen samples from a bumble bee (Hymenoptera: Apidae) collection show historic foraging on introduced and native plants in the South Island of New Zealand

**DOI:** 10.1371/journal.pone.0278860

**Published:** 2022-12-30

**Authors:** Mary Knowles, Xun Li, Carlos Lehnebach, Philip Lester, Julia Kasper

**Affiliations:** 1 School of Biological Sciences, Victoria University of Wellington, Wellington, New Zealand; 2 Institute of Geological and Nuclear Sciences, Lower Hutt, New Zealand; 3 Museum of New Zealand Te Papa Tongarewa, Wellington, New Zealand; Instituto Federal de Educacao Ciencia e Tecnologia Goiano - Campus Urutai, BRAZIL

## Abstract

Historic pollination networks are important to understand interactions between different plant and pollinator species, as well as to differentiate between causes and consequences of present insect population decline. Natural history collections in museums store biological proxy data, which is used to reconstruct historic pollination networks of bumble bees. Four bumble bee species (*Bombus terrestris*, *B*. *ruderatus*, *B*. *hortorum* and *B*. *subterraneus*) were introduced to Aotearoa New Zealand in 1885 specifically for pollination purposes. Pollen samples were collected from museum specimens of three of the four NZ species of bumble bee (excluding *B*. *subterraneus*) collected between 1954 and 1972 from 56 locations across the South Island, New Zealand. The most common plants identified on all three bumble bee species were *Calluna vulgaris* (heather), *Ulex* (gorse), *Cytisus* (broom), and *Trifolium repens* (white clover). However, all three bumble bee species also carried pollen from several native plants (e.g. *Arthropodium*, *Weinmannia*, *Plagianthus*, *Quintinia*, *Veronica*, *Melicytus*) and potentially had been involved in the pollination of these species. This study adds new plant species known to be foraged upon by bumble bees in Aotearoa New Zealand. Further studies on pollination networks in New Zealand will help us understand any changes in host plant preferences over time and after the time period covered by this study.

## Introduction

Before humans arrived in Aotearoa New Zealand (NZ), forests covered 85 percent of the land [1] with an overabundance of small native white or pale flowers, where rewards such as nectar and pollen are freely available to any insect pollinator or floral visitor [2]. These floral traits are commonly observed in plants with a generalist pollination syndrome known as “small bee syndrome” which have corresponding short tongue length [3]. It is believed that in NZ, this syndrome evolved as a response to a diverse but unspecialised native pollinator assemblage that is dominated by flies, beetles and solitary bees (27 endemic species) [3]. Forest cover significantly reduced after Europeans arrived in 1840. In the South Island of NZ, native forest was mainly restricted to the West Coast while the Canterbury and Otago regions were converted to tussocks and grasslands [1]. Pre-human times, New Zealand’s soils predominantly evolved under forest cover with low levels of nitrogen, phosphorus and sulphur and therefore need to have nutrients added for crops and pasture after land conversion [1]. Red clover (*Trifolium pratense*) and lucerne (*Medicago sativa*) were brought to New Zealand where they have become important nitrogen fixers and require heavy insects in large numbers to pollinate the flowers [4].

Therefore, four species of bumble bees, *Bombus terrestris* (Linnaeus, 1758), *Bombus ruderatus* (Fabricius, 1775), *Bombus hortorum* (Linnaeus, 1761), and *Bombus subterraneus* (Linnaeus, 1758), were introduced to New Zealand in 1885 in an effort to improve agricultural yields [4–6]. This was the first time in the world that an insect had been deliberately introduced to increase the yield of a specific crop (red clover) [7]. Today, bumble bees are important pollinators for kiwifruit (*Actinidia deliciosa*), blueberries (*Vaccinium corymbosum*), lucerne (*Medicago sativa*), red and white clover (*Trifolium pratense*, *Trifolium repens*), and greenhouse tomatoes [8], all introduced cultivars. Three of the four bumble bees in NZ are long-tongued (*Bombus ruderatus*, *Bombus hortorum* and *Bombus subterraneus*) with a preference for flowers with long corollas such as red clover, while *Bombus terrestris* is short-tongued and is a more efficient pollinator of flowers with short corollas such as lucerne. It has been suggested these bee species might compete with native pollinators for pollen resources [9] but there is little evidence for this in New Zealand [10].

Natural history collections contain valuable biological information to interpret the past [11]. Due to the decay-resistant exine (outer layer) of pollen, collection specimens contain valuable information of historical pollinators host plant use, given that pollen is preserved attached to zoological specimens [12]. Previous studies have used historic pollen samples from bee collections to identify population dynamics and pollination networks from the past (Kleijn & Raemakers, 2008; Colla et al., 2012; Scheper et al., 2014; Gous et al., 2015) [12–15].

In this study, we examine preserved pollen on bumble bee specimens from the Gurr Collection, held at the Museum of New Zealand Te Papa Tongarewa (MONZ) to understand historical bumble bee visitation of introduced and native plants in the South Island, NZ.

## Methods

**Fig 1:**
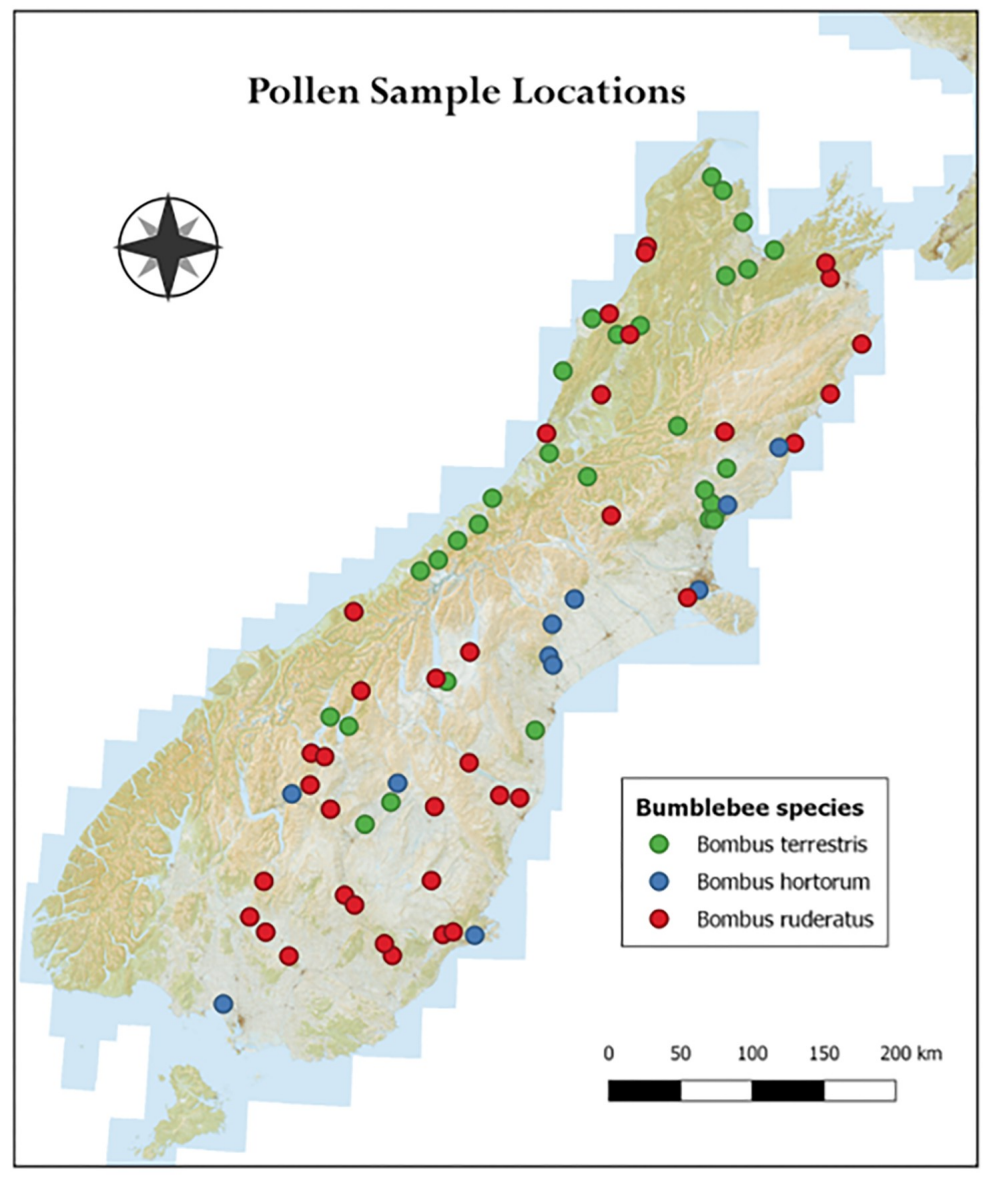
Map of the South Island with sample localities of bumblebees by L. Gurr between 1959 and 1972.

The Gurr collection has been used as a model in this study, providing information on pollination networks through time and space in New Zealand. Lou Gurr, an entomologist, undertook early surveys on bumble bee distribution in New Zealand and established populations of *B*. *hortorum* and *B*. *subterraneus* in the North Island [4]. His collection is now part of MONZ’s entomology collection, the majority of which is of three bumble bee species (*Bombus terrestris*, *Bombus ruderatus*, and *Bombus hortorum*) collected from the South Island All *Bombus* specimens used for this study were collected by Lou Gurr between 1954 and 1972 in the months of late spring to late summer, between November and March (see S1 Table). The location of the sample sites have been mapped using QGis (Open Source Geographic Information System) Version 3.16.1 LRT Hannover (Fig 1). No additional detailed information on Gurr’s collection methods can be found. The reasons for the lack of *Bombus subterraneus* in the collection is unknown although this species has always been relatively rare in New Zealand with a restricted distribution and short period of activity making observations rare [6]. A descriptive analysis was pursued to gain broad understanding of the collection, and samples were selected covering a spread across all parameters. Therefore, sample sizes within each parameter were not sufficient for statistical analysis or comparison between seasons or locations.

A total of 56 pollen samples were removed from three species of bumble bees found in this collection: *Bombus terrestris* (23 samples), *Bombus ruderatus* (25 samples), and *Bombus hortorum* (eight samples). Pollen was removed from dry pinned bee specimens using a fine brush or a scalpel [16] before being stored in 70% alcohol.

The samples were then processed using Erdtman’s acetolysis method [17]. Acetolysis is a chemical treatment routinely used to remove pollen protoplasm and clear the pollen cell wall, making it more suitable for morphological study and botanical identification. The sample was transferred from the storage vial into a 15 ml centrifuge tube and topped up with deionized water. After centrifuging at 2,500 rpm for 3 minutes, the sample residue was washed again in glacial acetic acid, and then heated for 4 minutes at 95–97°C in a 9:1 mixture of acetic anhydride and sulphuric acid. The residue was then washed in glacial acetic acid, followed by two water washes.

For optical microscope examination, a small quantity of subsample residue material was added to a drop of melted glycerine jelly on a heated microscope slide for slide making. The jelly contained a small quantity of red safranin stain to improve optical contrast of pollen cell wall. The slide was examined under a Zeiss A2 microscope at visual magnifications of 500 to 1250 times. Pollen identification was based on [18], online databases and GNS Science pollen reference collection, along with the analysts’ own experience.

Pollen taxa were not always identified to a species level (S1 Table). If the identification was above species levels it was not always possible to determine if the visited plant was an introduced or a native one. In this case, the plant species was labelled as ‘undefined’. The abundance of each plant species was not measured. The data was analysed in R Version 4.2.1 (2022-06-23) with optimal community structure and membership determined with the package “igraph” 1.3.4 (2022-07-19) [19, 20].

## Results

**Fig 2:**
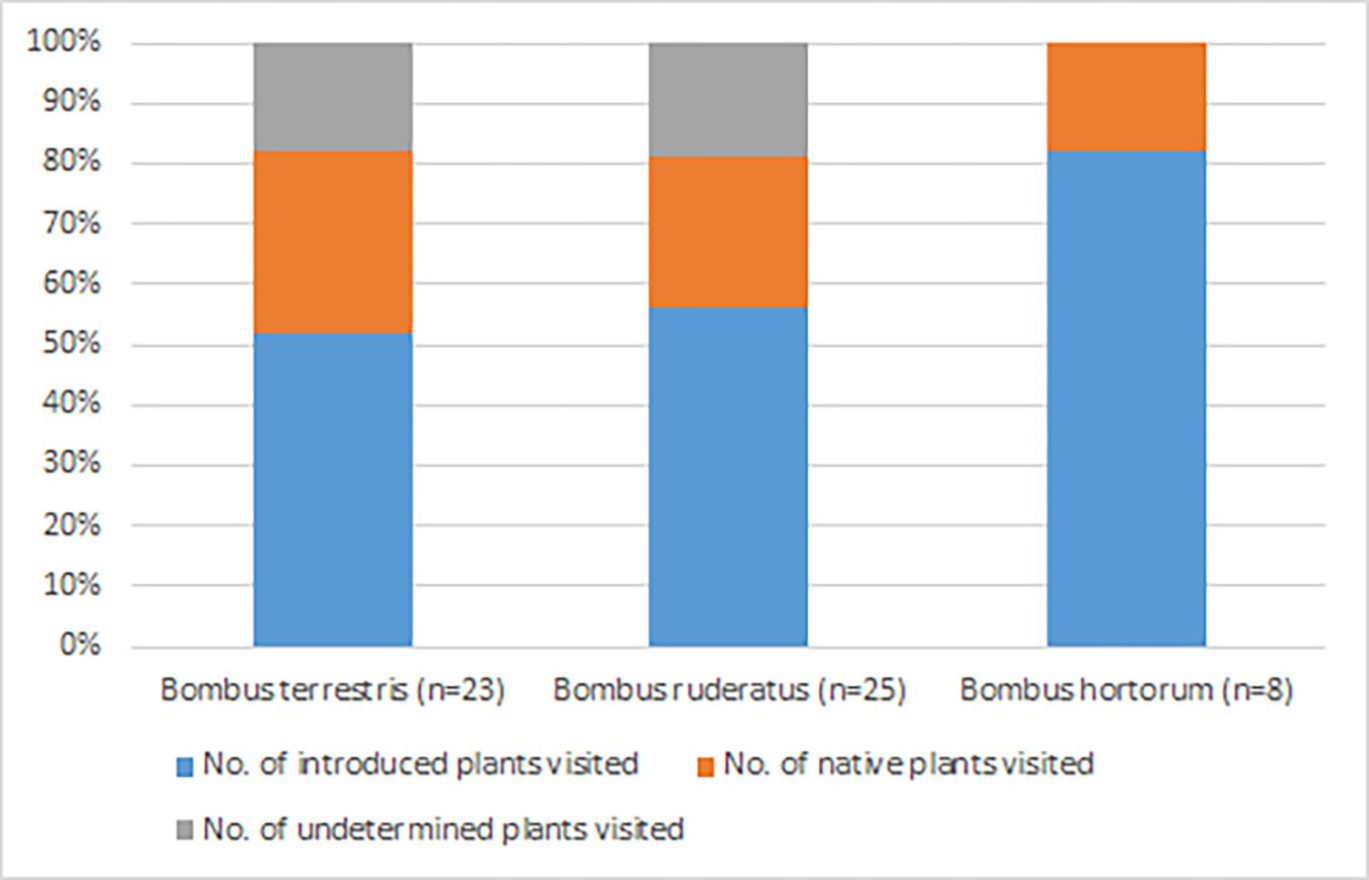
Quantitative comparison of introduced and native plants visited by the three bumble bee species *Bombus terrestris*, *Bonus ruderatus*, and *Bombus hortorum*.

**Fig 3:**
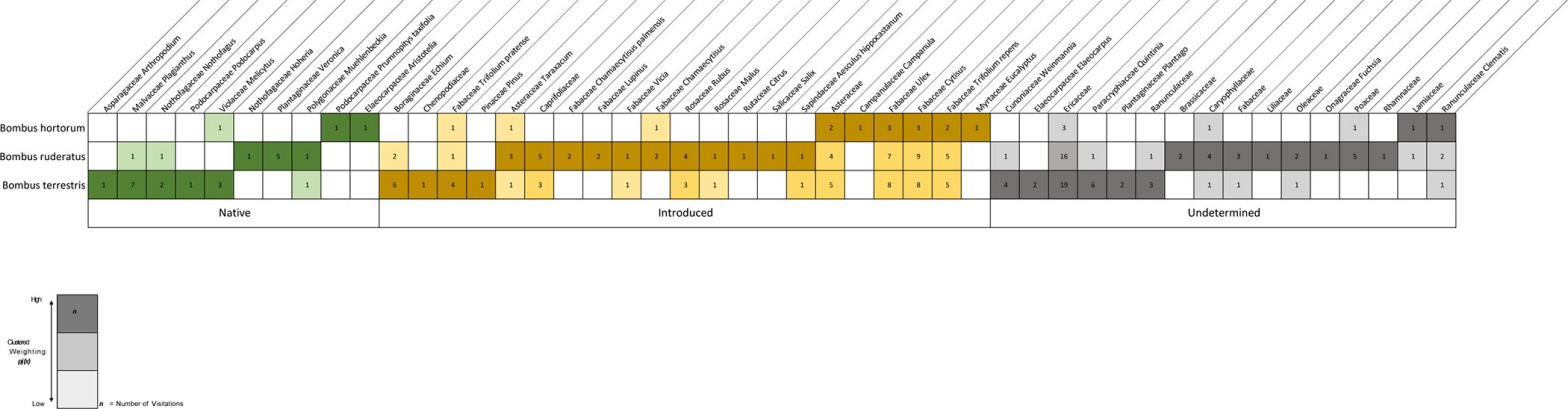
Weighted incidence matrix, showing clusters of plant species (pollen) found on the three bumble bee species *Bombus terrestris*, *Bonus ruderatus*, and *Bombus hortorum*, sorted by native, introduced and undetermined. Community structure determined by maximal modularity score.

Our analysis of pollen from these historic bumble bee specimens identified 49 families, 34 genera, and six species of plants were identified from the 56 pollen samples taken from the three species of bumble bee (S1 Table). All three bumble bee species carried a larger number of introduced plant species than of native plants, but in a different ratio (Fig 2). Of all three bumble bee species, *B*. *terrestris* had a higher mean number of native plant species in pollen samples than the other two species (Table 1). The species with native plant pollen were mainly samples on the West Coast (S2 Table). The most common plants identified on all three bumble bee species were *Calluna vulgaris* (heather), *Ulex* (gorse), *Cytisus* (broom), and *Trifolium repens* (white clover). Of the native plant species, only *B*. *ruderatus* carried *Veronica* (five occurrences), while *B*. *terrestris* mostly carried *Plagianthus* (Fig 3).

**Table 1:** The mean number (and range) of plant species in each pollen sample by bee species, and the mean number of native plants in each pollen sample by bee species.

Species	Mean number (and range) of plant species	Mean number (and range) of native plant species in each sample.
*B*. *terrestris*	4.18 (8)	1.17 (4)
*B*. *ruderatus*	4.08 (8)	0.44 (2)
*B*. *hortorum*	3.25 (5)	0.38 (2)

The plant species identified from each bumble bee species are listed below.

28 plant species were identified on *Bombus terrestris* (23 samples). Of which, 14 were introduced (*Taraxacum* (dandelion), *Echium* (borages), Caprifoliaceae (genus undefined), Caryophyllaceae (pinks), Chenopodiaceae, *Calluna vulgaris* (heather), *Ulex* (gorse), *Cytisus* (broom), *Trifolium repens* (White clover), *Trifolium pratense* (red clover), *Lupinus* (lupins), *Pinus* (pine), *Rubus fruticosus* (brambles), *Malus* (apples)), nine were native (*Arthropodium* (rengarenga), *Elaeocarpus*, *Weinmannia*, *Plagianthus* (manatu/ribbonwood), *Nothofagus* (tawhairauriki/Southern beeches), *Quintinia* (tawheowheo), *Podocarpus sp* (plum pine), *Muehlenbeckia*, *Melicytus* (māhoe/whiteywood)), and five were of undefined status (Ericaceae (heaths), Oleaceae (olives), *Plantago* (plantain), Ranunculaceae (buttercups), *Clematis* (leather flower)).

36 plant species were identified on *Bombus ruderatus* (25 samples). Of which, 18 were introduced (*Taraxacum* (dandelion), *Echium* (borages), *Campanula* (bellflowers), Caprifoliaceae (honeysuckle), Caryophyllaceae (pinks), *Calluna vulgaris* (heather), *Ulex* (gorse), *Cytisus* (broom), *Trifolium repens* (white clover), *Trifolium pratense* (red clover), *Chamaecytisus palmensis* (tree lucerne), *Vicia* (vetches), Poaceae (genus undefined), *Rubus* (brambles), *Malus* (apples), *Citrus* (citrus), *Salix* (willow), *Aesculus hippocastanum* (horse chestnut)), nine were native (*Arthropodium* (rengarenga), *Weinmannia* (kamahi), *Plagianthus* (manatu/ribbonwood), *Hoheria* (houhere/lacebark), *Nothofagus* (tawhairauriki/Southern beeches), *Quintinia* (tawheowheo), *Veronica*, *Muehlenbeckia*, *Melicytus* (māhoe/whiteywood)), and nine were of undefined status (Asteraceae, Brassicaceae (mustards), Lamiaceae (mints), Liliaceae (lilies), Oleaceae, Fabaceae, *Fuchsia*, Ranunculaceae, Rhamnaceae).

17 plant species were identified on *Bombus hortorum* (eight samples). Of which 12 were introduced *Taracaxum* (dandelion), *Echium* (borages), *Campanula* (bellflowers), *Calluna vulgaris* (heather), *Ulex* (gorse), *Cytisus* (broom), *Trifolium repens* (white clover), *Trifolium pratense* (red clover), *Chamaecytisus palmensis* (tree lucerne), *Chamaecytisus* (lucerne), *Eucalyptus*, *Pinus* (pine)) three were native (*Aristotelia* (makomako/wineberry), *Prumnopitys taxifolia* (matai), and *Melicytus* (māhoe/whiteywood)), and two were of undefined status (Asteraceae and Poaceae (grass)).

## Discussion

The occurrence of pollen on a specimen does not necessarily imply visitation or pollination of the plant. Some of the pollen identified, such as *Pinus*, are wind pollinated plants that could have been passively collected by the bumble bee. Pollen could have also been passively collected through other pollinators dropping pollen from previously visited plants. Therefore, there is a possibility that the plant identified on the bumble bee specimens was not visited or pollinated. However, *B*. *terrestris* carried a greater number of native plant species than the other bumble bee species (Table 1). Native bees and *B*. *terrestris* have shorter tongues than the other *Bombus* species which may explain the higher proportion of native plants identified on *B*. *terrestris* [21]. Fabaceae plants were identified on all *Bombus* species, reflecting their closed-access flag blossoms that can only be opened by large bees [3].

Although all three bee species did carry pollen from native plant species and may have had a role in pollinating them, the majority of the pollen species were introduced plant species. Native bees and introduced bees in NZ have many differing characteristics that could account for the lower proportion of native plants identified on the bumble bee specimens such as size (bumblebees are larger than native bees, and all native bees are short tongued), diurnal activity (bumblebees forage earlier than native bees), flight temperature thresholds (bumblebees tolerate cold), floral resource requirements (bumble bees require abundant pollen and nectar), and population dynamics (bumble bees form small colonies while native bees are solitary) [3]. This result suggests that bumble bees could impose less competition pressure for food resources on native pollinators and supports previous studies that have shown bumble bee species have a preference for introduced European plant species [6, 9], but we lack detailed data on plant and pollen availability for this region at that time to confirm this trend.

However, the geographical and spatial distribution of the dataset used in this investigation, provide a good reference to further examine plant-pollinator interactions over time because they have undergone land-use change since the specimens were collected (1954–1972). Economic factors played a role in driving this land use change. High market prices for sheep during the Korean War wool boom in the 1950s resulted in farmers converting large areas into pasture. Sheep numbers increased rapidly from 33.86 million sheep in 1950 to 60.28 million in 1970 before peaking in 1982 at 70.2 million [22]. Therefore, the plant assemblages in this study likely reflect the large amount of land used for sheep farming. The end of this study’s time period marks an important shift in farming in New Zealand. With the change in government in 1984, farming subsidies were removed, and farmers had to increase efficiency and diversify. Since 1982, sheep numbers have reduced dramatically, being replaced by forestry and dairy farming [23]. Therefore, today’s bumble bees may pollinate different plant assemblages. In north-western Europe, Kleijn and Raemakers [13] compared pollen loads of bumble bee entomology collections from the same area in 1950 and 2004/2005 and discovered significant differences in pollen composition between species associated with the spread of invasive plant species *Impatiens glandulifera*. The declining bee species was unable to switch to less preferred plant species, indicating the importance of not only host plant preferences for bee survival [12] but also the impact of land use change on these plant species. The land use change that has occurred at the localities from this study offers opportunities for future research on changes to bumble bee host plant preferences and offers information on historical plant assemblage in NZ.

If identification of pollen is assumed as visitation, this study adds to the number of different native plant species each bumble bee species has been recorded to forage upon. Firstly, *B*. *terrestris* was previously observed foraging on 46 native plant species [6], none of which included the eight native plant species identified in this study. Secondly, *B*. *ruderatus* was previously observed foraging on 1 native plant species (*Clianthus puniceus*) (Donovan, 2007). This study adds another nine native plant species that *B*. *ruderatus* is known to forage on. Thirdly, *B*. *hortorum* was previously observed foraging on three native plant species (*Fuchsia excorticata*, *Sophora microphylla*, and *Veronica* x*loganioides*) (Donovan, 2007). This study adds another three native plant species that *B*. *hortorum* is known to forage on.

This study reaffirms the potential of natural history collections to understand historic foraging patterns. Other work has shown the value of pollen analysis from museum collections of bees to help elucidate trends and context for bee declines. Sherper [12] identified pollen loads on wild bee specimens from entomological collections that were collected before the onset of their decline and used this host plant preference information with organism specific traits and other factors to identify main drivers for wild bee decline in the Netherlands. The decline of preferred host plant species and body size–an indirectly associated factor as larger species demand larger forage amounts–were negatively related to population trends, indicating food limitation as a key factor driving wild bee decline. Understanding host plant preferences of bumble bee species and other bee species in NZ will be important in future management of bee populations and plant-pollinator networks.

Further studies on pollination networks in New Zealand will help us understand any changes in host plant preferences over time and after the time period covered by this study. A better understanding of the floral diversity in these sites would help us understand pollen preference by these bee species. Furthermore, experiments under lab conditions in greenhouses will also help us understand the role of bumble bees as pollinators of native plants today [24, 25].

## Supporting information

S1 Table Overview of the pollen analysis collected off bumble bees from the Gurr collection at MONZ. (XLSX)

S2 Table Overview of the pollen analysis collected off bumble bees from the Gurr collection at MONZ sorted by three examined species (*Bombus terrestris*, *B*. *ruderatus* and *B*. *hortorum*). (DOCX)
