# Supplementary material for: Pollen samples from a bumble bee (Hymenoptera: Apidae) collection show historic foraging on introduced and native plants in the South Island of New Zealand
Source: PLoS One. 2022 Dec 30;17(12):e0278860. doi: 10.1371/journal.pone.0278860 (PMC9803242; doi:10.1371/journal.pone.0278860)
Supplement: S2 Table — (DOCX) [file pone.0278860.s002.docx]

Table S2:. Overview of the pollen analysis collected off bumble bees from the Gurr collection at MONZ sorted the sample sites into the regions (Marlborough, Canterbury, Otago, West Coast and Southland).

| **Family** | **Flower** | Marlborough | Canterbury | Otago | West Coast | Southland | **total** |
| --- | --- | --- | --- | --- | --- | --- | --- |
| [**Asparagaceae**](https://en.wikipedia.org/wiki/Asparagaceae) | *Arthropodium* |  |  | 1 | 1 |  | **2** |
| **Asteraceae** |  | 1 | 5 | 5 |  |  | **11** |
|  | *Taraxacum* |  | 2 | 2 | 1 |  | **5** |
| [**Boraginaceae**](https://en.wikipedia.org/wiki/Boraginaceae) | *Echium* |  | 2 | 3 | 4 |  | **9** |
| **Brassicaceae** |  |  | 1 |  |  | 1 | **2** |
| [**Campanulaceae**](https://www.google.com/search?rlz=1C1GCEV_en&sxsrf=ALeKk03WYOSxuhAkjFIi58uZtn_PtwmQ3Q:1610941439032&q=Campanulaceae&stick=H4sIAAAAAAAAAONgVuLUz9U3MM4wyzFZxMrrnJhbkJhXmpOYnJqYCgApwCrGHQAAAA&sa=X&ved=2ahUKEwiKsejByKTuAhXszTgGHbcHCPkQmxMoATAhegQIPxAD) | *Campanula* |  | 1 |  |  |  | **1** |
| **Caprifoliaceae** |  |  | 4 |  | 3 | 1 | **8** |
| **Caryophyllaceae** |  |  | 2 | 2 | 1 | 1 | **6** |
| **Chenopodiaceae** |  |  |  |  | 1 |  | **1** |
| [**Cunoniaceae**](https://en.wikipedia.org/wiki/Cunoniaceae) | *Weinmannia* |  |  |  | 6 |  | **6** |
| [**Elaeocarpaceae**](https://en.wikipedia.org/wiki/Elaeocarpaceae) | *Elaeocarpus* |  |  |  | 2 |  | **2** |
|  | *Aristotelia* |  | 1 |  |  |  | **1** |
| **Ericaceae** | *Calluna vulgaris* | 3 | 5 | 8 | 8 | 2 | **26** |
|  |  | 2 |  |  |  |  | **2** |
| **Fabaceae** | *Ulex* | 3 | 3 | 5 | 5 | 2 | **18** |
|  | *Cytisus* | 3 | 2 | 5 | 5 | 4 | **19** |
|  | *Trifolium repens* | 2 | 4 | 4 | 3 |  | **13** |
|  | *Trifolium pratense* | 3 | 2 | 1 |  |  | **6** |
|  |  | 1 |  | 1 | 1 | 1 | **4** |
|  | *Chamaecytisus palmensis* |  | 1 |  | 1 |  | **2** |
|  | *Lupinus* |  |  |  | 1 | 1 | **2** |
|  | *Vicia* |  | 1 | 1 |  |  | **2** |
|  | *Chamaecytisus* | 1 |  |  |  |  | **1** |
| **Lamiaceae** |  |  | 2 |  |  |  | **2** |
| **Liliaceae** |  |  |  | 1 |  |  | **1** |
| [**Malvaceae**](https://en.wikipedia.org/wiki/Malvaceae) | *Plagianthus* | 1 | 1 | 1 | 5 |  | **8** |
|  |  | 1 |  |  |  |  | **1** |
|  | *Hoheria* |  | 1 |  |  |  | **1** |
| [**Myrtaceae**](https://www.google.com/search?rlz=1C1GCEV_en&sxsrf=ALeKk01rhq2SIkAoLLPjnIsNmCA72oJXrg:1611018286491&q=Myrtaceae&stick=H4sIAAAAAAAAAONgVuLUz9U3MMxLskxfxMrpW1lUkpicmpgKAKb0cV8ZAAAA&sa=X&ved=2ahUKEwjsmcXl5qbuAhXlyzgGHRBcBmsQmxMoATAjegQIQBAD) | *Eucalyptus* |  |  |  |  | 1 | **1** |
| **Nothofagaceae** | *Nothofagus* |  |  |  | 2 | 1 | **3** |
| **Oleaceae** |  |  | 1 |  | 3 |  | **4** |
| [**Onagraceae**](https://www.google.com/search?rlz=1C1GCEV_en&sxsrf=ALeKk00KXtTzoP9CQ5dIz2mZz2Owke6T4A:1611018306493&q=Onagraceae&stick=H4sIAAAAAAAAAONgVuLUz9U3MK5KN85bxMrln5eYXpSYnJqYCgBMv4VKGgAAAA&sa=X&ved=2ahUKEwjLhIrv5qbuAhXfxjgGHYJHBHMQmxMoATAcegQIOBAD) | *Fuchsia* | 1 |  |  |  |  | **1** |
| [**Paracryphiaceae**](https://en.wikipedia.org/wiki/Paracryphiaceae) | *Quintinia* |  |  |  | 9 |  | **8** |
| [**Pinaceae**](https://en.wikipedia.org/wiki/Pinaceae) | *Pinus* |  |  | 1 | 1 |  | **2** |
| **Plantaginaceae** | *Veronica* | 1 | 1 | 3 |  |  | **5** |
|  | *Plantago* |  |  |  | 2 |  | **2** |
| **Poaceae** |  | 1 | 1 | 2 |  | 2 | **6** |
| **Podocarpaceae** | *Prumnopitys taxifolia* |  | 1 |  |  |  | **1** |
|  | *Podocarpus* |  |  |  | 1 |  | **1** |
| [**Polygonaceae**](https://www.google.com/search?rlz=1C1GCEV_en&sxsrf=ALeKk00ZkQ0UeOG-0PZH3g7yuMDb0cRrUw:1611018629070&q=Polygonaceae&stick=H4sIAAAAAAAAAONgVuLQz9U3MC3JzVjEyhOQn1OZnp-XmJyamAoAAoTq0BsAAAA&sa=X&ved=2ahUKEwjcyPKI6KbuAhUwyDgGHTJLCJkQmxMoATAfegQIIxAD) | *Muehlenbeckia* |  | 1 |  | 1 |  | **2** |
| **Ranunculaceae** |  | 1 |  |  | 4 |  | **5** |
|  | *Clematis* | 1 |  |  | 1 | 2 | **3** |
| **Rhamnaceae** |  |  |  | 1 |  |  | **1** |
| [**Rosaceae**](https://www.google.com/search?rlz=1C1GCEV_en&sxsrf=ALeKk03_8YZiqP3vP5YceuvIFOP1ob-0Yg:1611018511497&q=Rosaceae&stick=H4sIAAAAAAAAAONgVuLQz9U3MEvJzVjEyhGUX5yYnJqYCgC4UxFAFwAAAA&sa=X&ved=2ahUKEwjkoerQ56buAhU9zjgGHaLfB-gQmxMoATAdegQINxAD) | *Rubus* | 2 | 2 | 2 | 2 |  | **8** |
|  | *Malus* |  |  | 1 | 1 |  | **2** |
| **Rutaceae** | *Citrus* | 1 |  |  |  |  | **1** |
| [**Salicaceae**](https://www.google.com/search?rlz=1C1GCEV_en&sxsrf=ALeKk03e_1WXassMkra1Qbflt6T8oaArEw:1611022347408&q=Salicaceae&stick=H4sIAAAAAAAAAONgVuLUz9U3MMxLNipYxMoVnJiTmZyYnJqYCgD7hXAKGgAAAA&sa=X&ved=2ahUKEwjs7vf19abuAhX1wTgGHSd3CBcQmxMoATAeegQILhAD) | *Salix* |  | 1 |  |  |  | **1** |
| **Sapindaceae** | *Aesculus hippocastanum* |  | 1 |  | 1 |  | **2** |
| [**Violaceae**](https://en.wikipedia.org/wiki/Violaceae) | *Melicytus* |  | 1 |  | 3 |  | **4** |
